# Scapular bronchogenic cyst

**DOI:** 10.4103/0971-9261.44768

**Published:** 2008

**Authors:** Anjani Kumar Kundal, Noor Ullah Zargar, Anurag Krishna

**Affiliations:** Department of Pediatric Surgery, Sir Ganga Ram Hospital, Delhi - 110 060, India

**Keywords:** Bronchogenic cyst, sinus, tracheobronchial tree

## Abstract

We report a rare case of a 3-year-old male child with scapular bronchogenic cyst. The cyst was excised because of associated pain and discharge from the swelling. Till date, 64 cases of cutaneous bronchogenic cyst have been reported in the literature. Only 12 of these patients had lesion located in periscapular area. The treatment is surgical as it can undergo malignant transformation.

## INTRODUCTION

Bronchogenic cysts are rare lesions which originate from primitive tracheobronchial tree. They are primarily located in the thorax.[1] Extrathoracic locations may be either in the immediate vicinity of the thoracic cage (suprasternal notch) or more remote periscapular location which is extremely rare.[2]

## CASE REPORT

**Figure 1:**
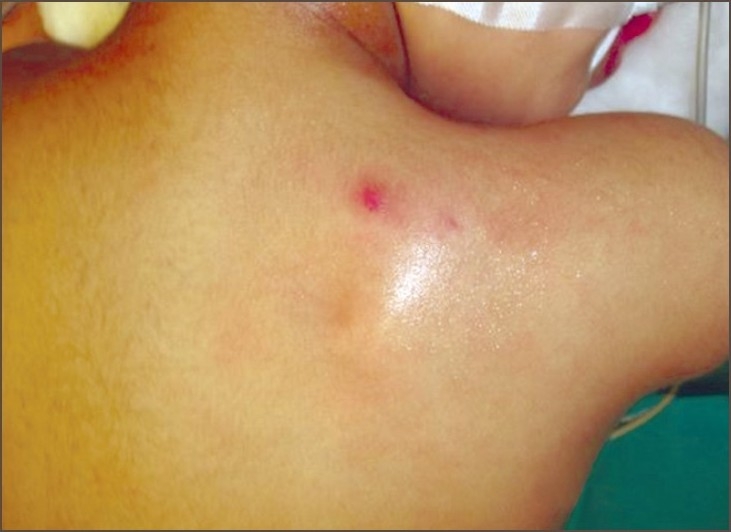
Swelling at the right side of scapular region

**Figure 2:**
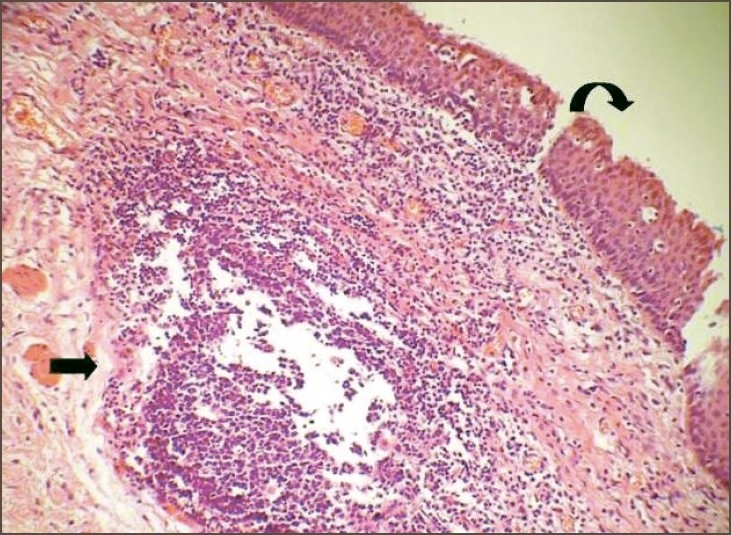
Cyst wall lined by ciliated columnar (bronchial) epithelium (curved arrow) and stratified squamous epithelium. Lymphoid follicles (straight arrow) present in the stroma (H&E, ×200)

A 3-year-old male child with swelling at the right side of scapular region was referred to our hospital [Figure 1]. Swelling was first noticed 2 years back. It was associated with pain and discharge. On examination, the swelling was 2 × 3 cm in size, tender, bony on palpation. The swelling could be moved separately from the scapula. A sinus opening was present on the top of the swelling. The child was administered antibiotics for a week. CT scan revealed a bony density lesion, posterior to the medial border of the right scapula with no communication with the scapula. A small nodular lesion with fat density was seen along with it. At surgery, the thick walled mass of 3 × 2 cm was totally excised along with the sinus opening. The pathological examination revealed a cystic lesion compatible with bronchogenic cyst [Figure 2]. No recurrence was noted at 6 months follow up.

## DISCUSSION

Bronchogenic cysts are rare with a prevalence range from 1 in 42000 to 1 in 68000.[3] Scapular location of bronchogenic cyst is extremely rare. They result from an abnormal budding of the tracheobronchial tree. During embryonic development, the primitive foregut arises in the third week of gestation and divides into dorsal portion, which elongates to form the esophagus and ventral portion, which differentiates into the tracheobronchial tree. Errors in the development of the ventral foregut will give rise to bronchogenic cysts.[4,5] It is possible that accessory buds from the tracheobronchial tree/primitive foregut may get excluded from the thorax and migrate in an unusual manner to lie in periscapular location. Other mechanism can be in situ development of the respiratory epithelium due to metaplasia of mature preexisting cutaneous tissue and primary anomalous differentiation (heterotopia) in the developing skin.[6] Cyst wall consists of hyaline cartilage, smooth muscle cells, elastic fibers, fibrous tissues, neural cells and seromucous glands.[2,7] Histologically, they are lined by pseudostratified ciliated columnar epithelium, which can rarely undergo malignant transformation.[8] Making preoperative diagnosis of bronchogenic cyst is very difficult. MRI may be useful.[9] Most bronchogenic cysts contain large amount of proteinaceous material and characteristically have high signal intensity on T1-weighted images. In Fraga's study, which is largest collection of patients with congenital bronchogenic cysts, 12 out of 30 patients (40%) were diagnosed before 4 years of age.[2] They can be diagnosed as slow growing masses or as draining sinuses. Mass over scapula is the most common complaint in scapular congenital bronchogenic cysts patients. The definitive treatment is total excision due to risk of infection and malignant degeneration, which has been reported in scapular bronchogenic cysts.[10]

In conclusion, the rare possibility of congenital bronchogenic cyst should be kept in mind while dealing with superficial scapular skin lesion. The diagnosis is histopathological in majority of cases.
